# Fragment-based Binding Efficiency Indices in Bioactive Molecular Design: A Computational Approach to BACE-1 Inhibitors 

**Published:** 2013

**Authors:** Nima Razzaghi-Asl, Ahmad Ebadi, Najmeh Edraki, Sara Shahabipour, Ramin Miri

**Affiliations:** a *Medicinal and Natural Products Chemistry Research Center, Shiraz University of Medical Sciences, Shiraz, Iran, PO Box 3288-71345.*; b *Department of Medicinal Chemistry, School of Pharmacy, Shiraz University of Medical Sciences, Shiraz, Iran, PO Box 1583-71345. *

**Keywords:** Alzheimer, BACE-1, Docking, Ligand efficiency

## Abstract

One of the most important targets in Alzheimer disease is Beta site amyloid precursor protein cleaving enzyme-1 (BACE-1). It is a membrane associated protein and is one of the main enzymes responsible for amyloid *β *(A*β*) production. Up to now, a considerable number of peptidic and non-peptidic inhibitors of BACE-1 have been developed. Recently, small molecule BACE-1 inhibitors have attracted the attention of scientists, because peptidic inhibitors have many pharmacokinetic problems. In the present study, several small molecule BACE-1 inhibitors were extracted from Brookhaven Protein Databank (PDB) and subjected to dissection analysis to achieve constructing fragments. Atom type, hybridization, and bond order were considered for generated constitutional fragments (simplified structures). AutoDock version 4.2 was applied to dock various chemical fragments into BACE-1 active site. The benefits of such studies have been well revealed in previous reports. On the basis of obtained binding affinities, fragment-based ligand efficiency (LE) indices were estimated. These theoretical binding efficiencies were applied to further elucidate the key structural features of BACE-1 inhibitors. Typical results of the study were elucidated and we suggested the ways these findings might be beneficial to guide rational bioactive molecular developments. Our study confirmed that the evaluation of ligand-receptor interactions in terms of ligand efficiency indices (binding energy per atom and pK_i _per MW) could be a helpful strategy in structure-based drug discovery (SBDD) strategies.

## Introduction

Alzheimer disease was diagnosed for the first time in a German patient named Alois Alzheimer (1). It is one of the diseases affecting a large number of people all over the world; being the most common cause of dementia in the elderly (1, 2). The amyloid cascade hypothesis proposes that aggregation of amyloid beta 40 and 42 (A*β*40 and A*β*42) oligopeptides followed by generation of neurotoxic plaques in brain as commonly occurring features in AD (3). Proteolytic cleavage of a large trans-membrane protein, amyloid precursor protein (APP), by two enzymes namely *β *and *γ*-secretases results in secretion of A*β*40 and A*β*42 peptides (4,5). *β*-secretase (Beta-site APP cleaving enzyme or BACE-1) is a type I membrane-associated aspartyl protease (3), which has been an attractive therapeutic target in AD due to the fact that it catalyzes the first step in A*β *production and is mainly expressed in brain. 

Therefore, development of specific inhibitors of these key proteases has been regarded as a major therapeutic target in AD treatment and many research groups have focused on development of beta-secretase inhibitors (6, 7). Nowadays, it is generally accepted that biologically potent compounds will not necessarily end in good drugs and that there are several critical parameters along the discovery process such as MW or partition coefficient responsible for the optimal pharmacological outputs (8). These simple property counting rules have been applied successfully to distinguish between drugs and non-drugs (9-11). 

Experimental measurement of binding affinities for huge molecular libraries is a time-consuming and non-economic process being a major bottle-neck in the field of drug discovery. To overcome this limitation, virtual screening techniques have found their usefulness in finding potential bioactive compounds prior to synthesis. The concept of interpreting ligand-receptor interaction in terms of the free energy per atom (ligand efficiency, LE) was first proposed by P. Andrews (12). Consequently several efficiency indices have been proposed by other groups (13). These ligand-based efficiency indices are now regarded as undeniable part of modern lead development strategies (14, 15). In this regard, the use of structure-based calculated binding free energies (16) instead of experimental binding affinities may become successful alternative for obtaining LEs. Estimated LEs may offer negligible time and, consequently, reduce time-consuming and expensive biochemical measurements. In an AutoDock based study, BACE-1 inhibitors were used to correlate the estimated and experimental LEs. Results showed an acceptable correlation among the data (17).

Availability of a significant amount of crystallographic data on Protein Data Bank (PDB, http://www.rcsb.org) has facilitated the performance of structure based drug discovery projects aiming at beta-secretase as a molecular target for Alzheimer disease. *Holo *X-ray crystallographic structures which bear cognate ligands are principal sources for this purpose (18).

In the present work, the PDB database was screened for potential small molecule BACE-1 inhibiting molecules and 83 structures were extracted for our structure-based study. Dissection analysis was performed on two-dimensional structures of the selected compounds. This shape-based analysis resulted in fragments, graph-based frameworks and side chains. Obtained fragments were subjected to docking simulations into BACE-1 active site and results were further discussed for binding energies and binding efficiency indices.

*Computational section*

Chemical structures of ligands under study were all extracted from *Holo *PDB files documented in PDB database (83 PDB codes). Our criterion for selected molecules considered chemical structures bearing 10 or less rotatable bonds after dissection into simplified structures (19). Molecular descriptors were calculated by Dragon software version 2.1 (20).

Flexible-ligand docking studies were performed using AutoDock 4.2 program (21). All the pre-processing steps for ligand and receptor crystallographic files were performed within WHAT IF server (European Molecular Laboratory Heidelberg, Germany) and AutoDock Tools 1.5.4 program (ADT) which has been released as an extension suite to the Python Molecular Viewer (22, 23). All hydrogens were properly added to the receptor PDB file using What if server. ADT program was used to merge non-polar hydrogens into carbon atoms of the receptor and Kollman charges were also assigned. For docked ligands, non-polar hydrogens were also added. Gasteiger charges assigned and torsions degrees of freedom allocated by ADT program. Chemical fragments were docked into BACE-1 active site holding their initial conformation unchanged. The Lamarckian Genetic Algorithm (LGA) was applied to model the interaction pattern between BACE-1 and inhibitors. For all docking procedures, 150 independent genetic algorithm (GA) runs were considered for each molecule under study. A maximum number of 5×10^6^ energy evaluations; 27000 maximum generations; a gene mutation rate of 0.02 and a crossover rate of 0.8 were used for Lamarckian genetic algorithm. The grid maps representing BACE-1 in the docking process were calculated using AutoGrid (part of the AutoDock package) with a grid spacing of 0.375 Å (roughly a quarter of the length of a carbon–carbon single bond). A grid of 60, 60, and 60 points in x, y, and z directions was centered on the center of mass of the active site of BACE-1 since the locations of the original ligands (cognate ligands) in the complex were known. Cluster analysis was performed on the docked results using a root mean square (RMS) tolerance of 2 Å. Schematic 2D representations of the ligand-receptor binding maps were generated using LIGPLOT (24). 

## Results and Discussion

*Dissection analysis of BACE-1 inhibitors*

**Figure 1:**
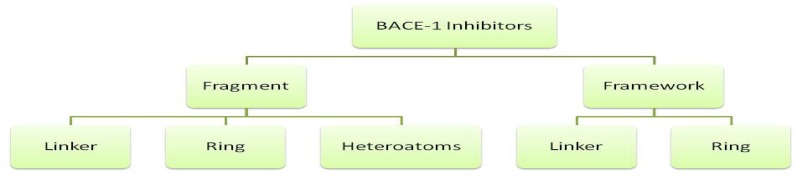
Hierarchical dissection of BACE-1 inhibitors

In order to better recognize the structural features of BACE1 inhibitors, we used a fragmentation method on the compounds originated from PDB database. BACE1 inhibitors were classified into several constructive units. Chemical structures and their fragments are depicted in a hierarchy model in Figure 1. The advantages of such dissection methods have been well established in previous reports (25). 

Frameworks are molecular descriptions on the basis of graph theory in which atoms and bonds are depicted as vertexes and edges of a graph ignoring atom types, atomic hybridizations, atomic charges and bond orders (26). In scaffolds, molecular properties such as atom type, atom hybridization and bond order are incorporated. Side chains are non-ring, non-linker atoms. Linker atoms are situated at the pathway connecting ring systems. One distinguished feature in our present study is that we considered linkers without any elimination of functional groups such as carbonyl moieties or other side branches. In this way, detailed structural features of linker groups maybe provided. PDB codes of BACE-1 inhibitors and related fragments are summarized in Table 1. 

**Table 1:** Calculated descriptors for BACE-1 inhibitors (PDB codes) and their fragments

**PDB code**	**HAC** ^a^	**HDs** ^b^	**HAs** ^c^	**MW**
	**ligand / fragment**	**ligand / fragment**	**ligand / fragment**	**ligand / fragment**
1TQF	40 / 28	4 / 1	9 / 6	571.7 / 395.1
1W51	39 / 28	3 / 3	7 / 4	531.7 / 374.2
2EWY	38 / 37	3 / 3	5 / 5	504.6 / 490.2
2F3F	34 / 19	5 / 3	8 / 3	514.7 / 297.1
2IQG	41 / 28	3 / 3	6 / 6	677.6 / 374.2
2OF0	19 / 17	1 / 1	4 / 4	265.3 / 237.1
2OHL	13 / 10	2 / 0	2 / 1	144.2 / 129.1
2OHK	13 / 10	2 / 0	2 / 1	144.2 / 129.1
2OHM	15 / 14	3 / 1	3 / 2	199.3 / 184.1
2OHN	15 / 13	1 / 1	1 / 1	193.3 / 175.1
2OHP	21 / 17	3 / 1	3 / 2	237.3 / 222.1
2OHQ	23 / 20	2 / 0	3 / 1	304.4 / 259.1
2OHR	23 / 20	3 / 1	4 / 3	276.3 / 261.1
2OHS	24 / 20	3 / 1	5 / 3	306.4 / 261.1
2OHT	24 / 23	4 / 2	4 / 3	314.4 / 299.1
2OHU	32 / 31	4 / 2	6 / 5	421.5 / 406.2
2P83	44 / 28	5 / 3	10 / 4	610.7 / 374.2
2Q11	31 / 30	2 / 0	6/ 5	288.4 / 403.2
2Q15	37 / 36	2 / 0	6 / 5	496.6 / 487.3
2QK5	42 / 28	3 / 3	7 / 4	581.7 / 374.2
2QMD	44 / 32	3 / 3	7 / 5	607.7 / 430.2
2QMF	43 / 31	3 / 3	7 / 5	593.7 / 416.2
2QP8	40 / 25	3 / 3	7 / 4	559.7 / 338.2
2QU2	24 / 17	4 / 1	5 / 1	318.4 / 219.1
2QU3	29 / 17	4 / 0	5 / 0	427.9 / 236.1
2VA5	19 / 17	4 / 2	5 / 3	254.3 / 223.1
2VA6	24 / 18	2 / 1	5 / 2	323.4 / 234.1
2VA7	26 / 18	2 / 1	5 / 2	341.4 / 234.1
2WF4	40 / 35	2 / 4	9 / 7	554.7 / 492.2
2WJO	35 / 34	3 / 1	4 / 5	473.6 / 458.2
2ZDZ	35 / 24	4 / 1	7 / 2	486.9 / 311.1
2ZE1	34 / 26	5 / 2	7 / 3	516.4 / 338.1
2ZJH	20 / 13	1 / 0	3 / 1	292.4 / 175.1
2ZJI	24 / 13	1 / 0	5 / 1	352.5 / 175.1
2ZJJ	20 / 13	2 / 1	4 / 2	297.4 / 176.1
2ZJK	21 / 13	2 / 1	4 / 2	311.4 / 176.1
2ZJL	15 / 13	1 / 0	5 / 1	431.4 / 175.1
2ZJM	35 / 24	3 / 1	9 / 4	526.1 / 324.1
2ZJN	36 / 24	3 / 1	9 / 4	540.1 / 324.1
3BRA	10 / 6	3 / 0	2 / 0	137.2 / 78.1
3BUF	11 / 6	3 / 0	2 / 0	151.2 / 78.1
3BUG	12 / 6	3 / 0	2 / 0	165.2 / 78.1
3BUH	16 / 12	3 / 0	2 / 0	219.3 / 160.1
3CIB	44 / 32	3 / 3	6 / 4	605.8 / 428.2
3CIC	45 / 32	3 / 3	8 / 5	620.7 / 427.2
3CID	44 / 31	3 / 3	8 / 5	606.7 / 413.2
3DV5	35 / 27	3 / 4	6 / 4	487.7 / 372.2
3EXO	25 / 19	2 / 1	6 / 3	340.4 / 247.1
3FKT	36 / 30	2 / 1	7 / 4	491.6 / 400.2
3H0B	28 / 19	3 / 1	8 / 2	386.4 / 248.1
3HVG	11 / 6	3 / 1	4 / 2	153.2 / 80.1
3HW1	16 / 15	2 / 0	4 / 3	214.3 / 199.1
3IGB	22 / 19	2 / 0	4 / 3	418.5 / 275.1
3IN3	26 / 23	2 / 1	6 / 4	343.4 / 298.1
3IN4	30 / 23	2 / 1	7 / 5	400.5 / 299.1
3IND	24 / 21	2 / 1	4 / 2	323.5 / 278.2
3INE	27 / 21	2 / 1	5 / 2	367.5 / 278.2
3INF	29 / 23	2 / 1	6 / 3	386.4 / 297.1
3INH	32 / 23	2 / 1	7 / 4	445.4 / 298.1
3IVH	34 / 28	3 / 3	4 / 4	472.6 / 380.2
3IVI	37 / 31	4 / 4	6 / 6	510.6 / 418.2
3KMX	17 / 6	3 / 0	3 / 0	272.8 / 78.1
3KMY	16 / 14	2 / 0	2 / 1	232.7 / 183.1
3KN0	24 / 23	2 / 0	4 / 3	321.4 / 306.2
3L3A	32 / 24	2 / 0	5 / 2	442.9 / 310.2
3L5B	20 / 12	2 / 1	4 / 2	293.8 / 158.1
3L5C	32 / 23	4 / 3	8 / 5	432.3 / 306.1
3L5D	28 / 12	4 / 1	7 / 2	387.5 / 158.1
3L5E	39 / 33	3 / 2	7 / 4	535.8 / 446.3
3L5F	23 / 20	2 / 2	4 / 2	319.5 / 274.2
3L38	33 / 31	2 / 0	6 / 5	453.9 / 404.1
3L58	42 / 28	3 / 3	7 / 4	581.7 / 374.2
3L59	17 / 12	2 / 1	4 / 2	251.7 / 158.1
3LHG	28 / 23	2 / 1	5 / 3	378.4 / 297.1
3LNK	45 / 33	3 / 3	7 / 6	620.7 / 443.2
3LPI	46 / 34	3 / 3	7 / 7	656.8 / 479.2
3LPJ	44 / 32	3 / 3	7 / 6	606.7 / 429.2
3MSJ	15 / 9	3 / 1	4 / 2	225.7 / 118.1
3MSK	24 / 22	2 / 0	5 / 4	348.9 / 299.2
3MSL	25 / 23	3 / 1	5 / 4	347.9 / 313.2
3MSM	28 / 26	3 / 1	5 / 4	400.9 / 351.2
3PI5	29 / 24	3 / 1	5 / 1	480.4 / 337.2
3QBH	38 / 27	3 / 2	8 / 3	544.7 / 380.2

^a^HAC: Heavy atom count (number of atoms other than hydrogen). ^b^HDs: Hydrogen donors (Ns+Os).^c^HAs: Hydrogen acceptors (NHs+OHs)

**Figure 2:**
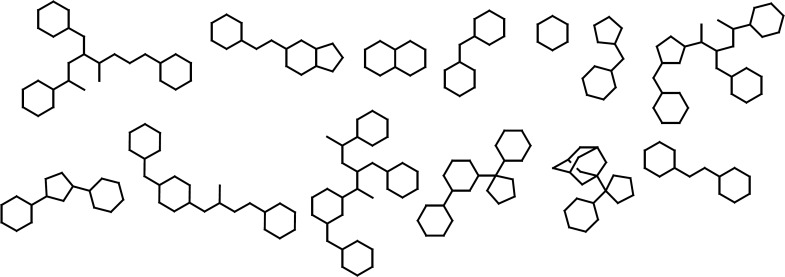
Abundant Graph-based frameworks of evaluated BACE-1 inhibitors documented in the Brookhaven protein databank as a result of dissection analysis

**Figure 3:**
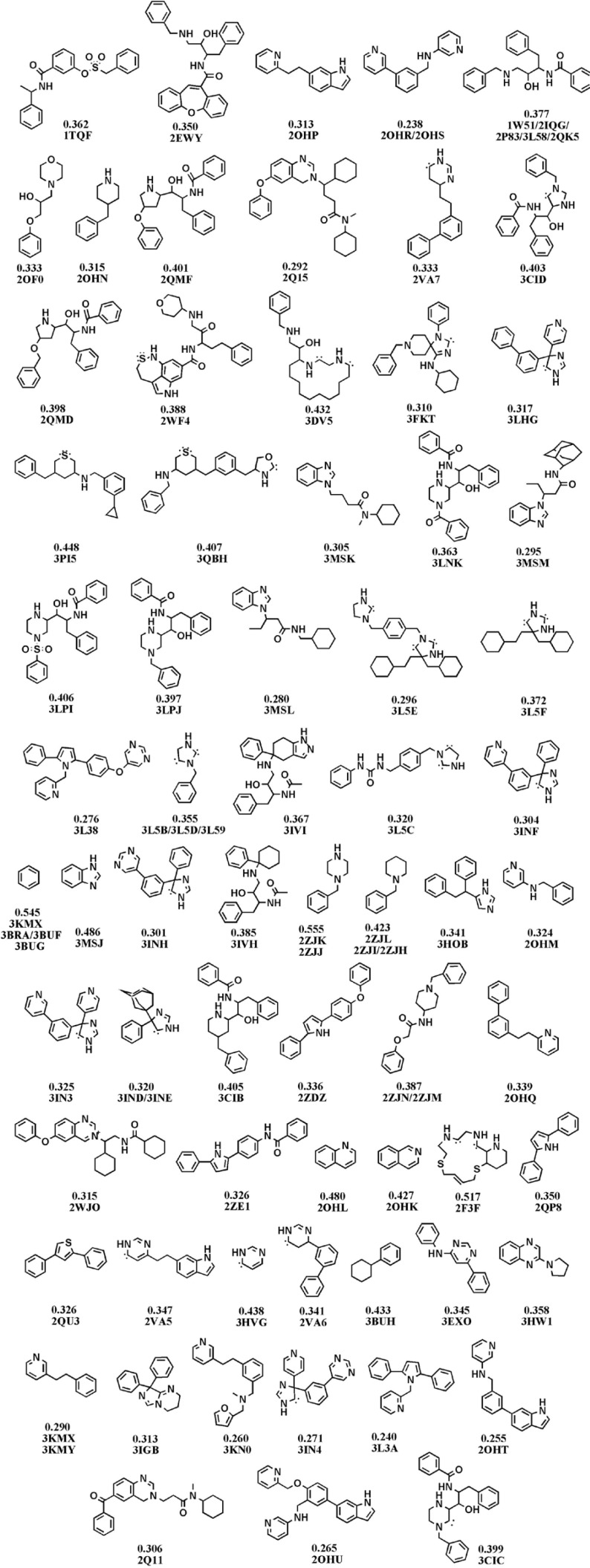
Fragments of BACE-1 inhibitors documented in the Brookhaven protein databank as a result of dissection analysis (Each structure is designated by attributed PDB code(s) and LE values in kcal.mol^-1^ unit).

It was crucial to retain the atomic hybridizations within simplified structures when side chains were removed, therefore, dot pairs were considered next to the sp^2 ^atoms to designate the related π electrons (25). No acyclic molecule was found and all structures under study possessed at least one ring system. The thing which is worth noting is the definition of ring and cycle. The cycles alone or fused to other cycles represent a unit ring system within each molecule. Graph-based frameworks and simplified structures of small molecule BACE1 inhibitors are shown in Figures 2 and 3. 

As it would be expected, classification scheme based on frameworks represented less diversity compared to the molecular scaffolds, because atom types, hybridizations and bond orders were not incorporated. Most of the evaluated BACE-1 inhibitors could be represented with 13 independent frameworks (Figure 2). 

*Ligand efficiency indices*

Ligand efficiency index can be simply calculated using the Equation 1 (12): 

$E=\frac{{-\mathrm{\Delta G}}_{b}}{\mathrm{HAC}}$                    (1)

HAC stands for heavy atom count. Concept ‘efficiency’ of a ligand could be a useful parameter in considering the real potency of a compound and hence optimizing fragments (27). Molecules that exhibit a distinct potency with fewer heavy atoms are by definition more efficient (28). Accordingly, obtained simplified chemical structures were re-ranked depending on their ligand efficiency values to be evaluated more sensibly. Regarding the ligand efficiencies, C.A. Zapatero *et al*. postulated that molecular weights are prior to the number of non-hydrogen atoms in considering the contribution of heteroatoms from different rows of the periodic table (13). These authors suggested a modified efficiency value entitled “binding efficiency index” (BEI). This index could be easily estimated from Equation 2: 

$\mathrm{BEI}=\frac{{\mathrm{pK}}_{i}}{\mathrm{MW}(\mathrm{KDa})}$                    (Equation 2)

The importance of BEI can be emphasized as an increase in molecular weight at the clinical candidate step, which is regarded as an undeniable paradox with a common trend towards lower MWs and better pharmacokinetic profiles in marketable drugs (29). 

Molecular fragments were subjected to validated docking study into BACE1 active site. Auto Dock scores were used to rank docked fragments. Experimental biological data (*In-vitro *FRET Assay) were correlated well with our estimated free binding energies (30). Calculated free binding energies, LE and BEI values for our studied fragment are introduced in Table 2. 

**Table 2:** Docked binding energies, ligand efficiencies (LEs) and binding energy indices (BEIs) of fragments derived from crystallographic BACE-1 inhibitors

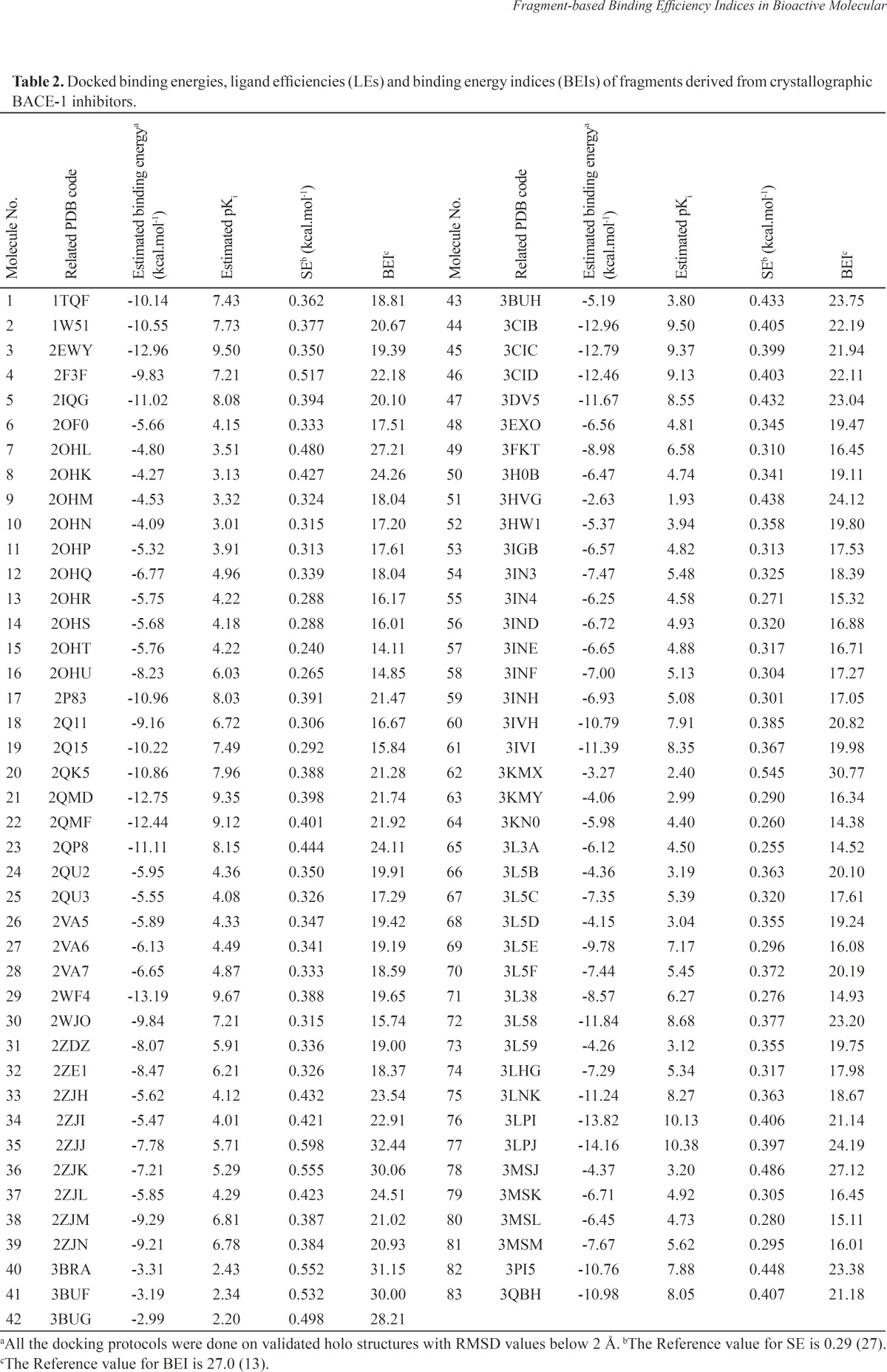

**Figure 4:**
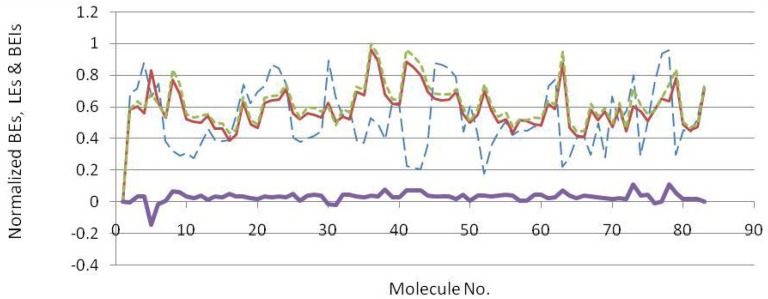
Normalized docking binding energies (dash blue line), ligand efficiencies (solid narrow red line), binding efficiency indices (dotted green line) and ΔEs (BEI-LE) (Solid thick violet line) for docked BACE-1 inhibiting fragments (molecule numbers are attributed to the order in Table 2).

Docked energies (BEs) and related efficiency indices were normalized and plotted (Figure 4). The distribution of LE and BEI patterns were relatively the same while estimated binding energies followed a different pattern (Figure 4). According to the plots, some useful hints may be concluded. 

- The analogous distribution pattern of LE and BEI efficiency plots may be attributed to the proximity of varying scaffold heteroatoms (N, O and carbon) in a periodic table. 

- As would be expected, the biggest difference between LEs and BEIs occurred in the case of BACE-1 inhibiting fragments bearing two sulfur atoms (2F3F, No.4 in Figure 4). Different BEI and LE values could be predicted in scaffolds possessing heavier heteroatoms such as sulfur or phosphorous in ring(s) or spacer(s). 

**Figure 5:**
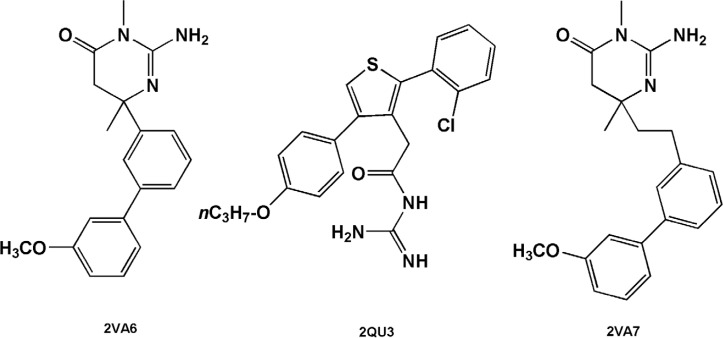
BACE-1 inhibitors including diphenylthiophene (2QU3) and biphenyl dihydropyrimidinone (2VA6 & 2VA7) scaffolds

Estimated binding energies could not be often used to define priorities among bioactive compounds while meaningful priorities may be established regarding LEs or BEIs. An appropriate example can be observed in the case of isophthalamide (3CIB, No.44 in Figure 4) and phenoxepine (2EWY, No.3 in Figure 4) derived fragments. In our docking study, these molecules exhibited identical binding energies to the BACE-1 active site, however, higher BEI and LE scores were assigned to isophthalamides. Crystallographic ligands identified by 2VA6, 2QU3 and 2VA7 codes in PDB database are potent BACE-1 inhibitors in micromolar range (http://www.pdb.org). These molecules are based on the diphenylthiophene (2QU3, Figure 5) and biphenyl dihydropyrimidinone (2VA6 & 2VA7, Figure 5) scaffolds exhibiting relatively low interaction energies in our docking evaluation while showing better BEI and LE indices (Table 3, Figure 4). As another example, benzimidazole fragment (3MSJ, No.78 in Figure 4) was found to be a weak BACE-1 inhibitor considering its docked binding energy. However, focus on the BEI value (and to the less extent LE value) demonstrated the efficiency of this scaffold for possible lead development strategies. 

**Table 3:** Estimated binding/efficiency scores of the isomeric fragments (BACE-1 inhibitors).

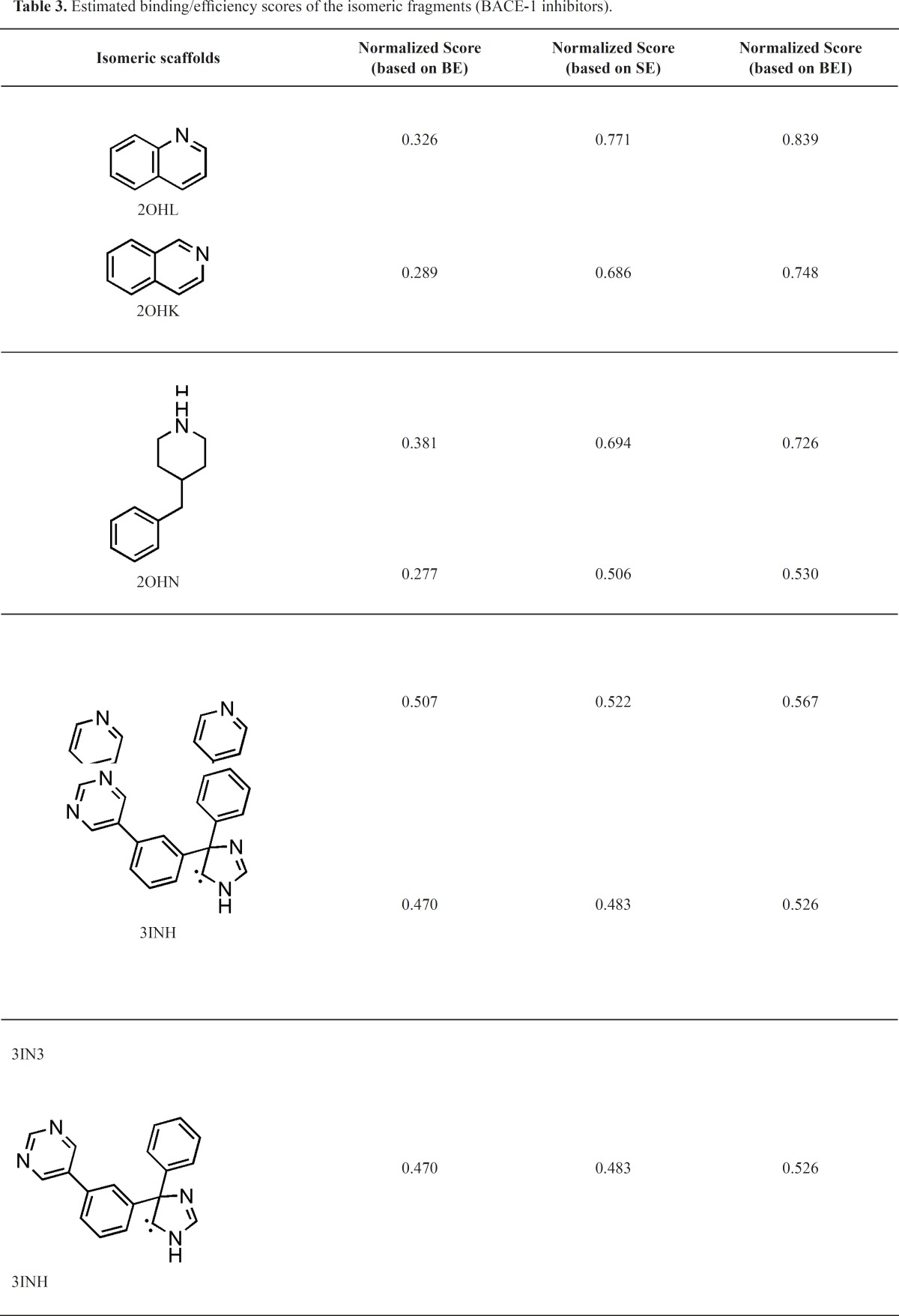

In some cases, high-ranked binding energies, LEs and BEIs were obtained. Chemical fragments possessing this feature provide optimum cases in developing potent BACE1 inhibitors. Some related examples included 3PI5, 3L5C, 3KN0, 2OHT, 3L3A, 2ZE1, 2ZDZ, 2QP8, 3IN4, 2ZJN and 3MSL based fragments. Our results revealed that benzylpiperazines (2ZJJ and 2ZJK) had higher docked energies among evaluated compounds. 3LPJ derived fragment exhibited highest estimated docked energy and also good efficiency indices. 3LPJ derived fragment may be an efficient scaffold to develop potent BACE-1 inhibitors (Table 2). This particular case can also be assessed from another aspect. Based on docking outputs, isophthalamides and benzylpiperazines have been found to be efficient BACE-1 inhibitors and one of the most potent compounds in PDB database (3LPJ) is a chimeric (hybrid) molecule comprising of these two building blocks. Considering efficient building blocks, similar trends may be extended for further hybrid potent BACE-1 inhibiting structures. 

**Figure 6:**
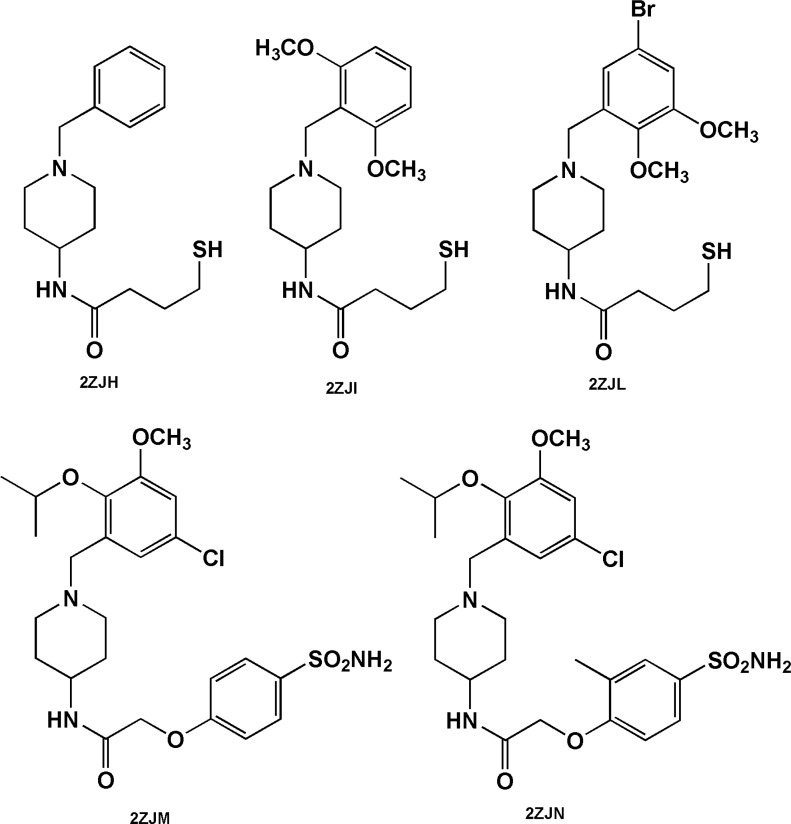
Benzylpiperidine-based BACE-1 inhibitors including mercaptobutanamide (2ZJH, 2ZJI and 2ZJL) and phenoxyacetamide (2ZJN, 2ZJM) side chains

The case of 4-amino-benzylpiperidines is noticeable. Chemical structures bearing mercaptobutanamide side chain exhibit higher efficiencies and lower docked energies (2ZJH, 2ZJI and 2ZJL, Figure 6) while 4-amino-benzylpiperidines comprising phenoxyacetamide side chain (2ZJN, 2ZJM, Figure 6) were found to be more potent BACE-1 inhibitors (retaining acceptable BEI and LE values, Table 3). 

*Isosterism and ligand efficiency values*

**Figure 7:**
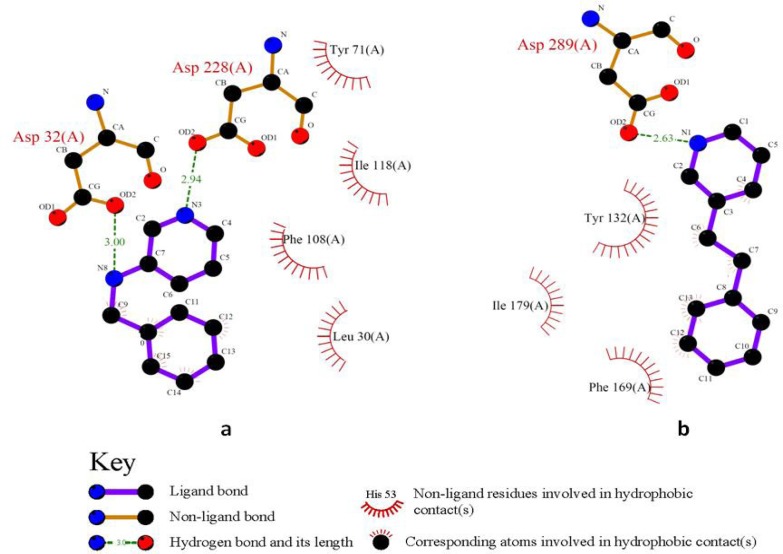
2D schematic interactions of 2OHM a: and 3KMY b: derived fragments with BACE-1 active site generated by LIGPLOT

2,5-diphenylpyrrole based fragment (2QU2) proved to be a little more potent and efficient than 2,4-diphenylthiophene (2QU3) (Table 3). Similar situations were observed for the other isosteric molecules (3INH and 3LHG; 3IN3 and 3IN4). Some isosteric substitutions provided potential hydrogen donor/acceptor sites for binding to the receptor (2OHM and 3KMY, 3LPJ and 3CIB, Figure 7). Due to the proximity of carbon and nitrogen atoms in a periodic table (one unit of mass difference) higher efficiency values would be expected for these fragments. In some cases, these isosteric replacements cause significant enhancements in potency results (2ZJK and 2ZJL). 

*Constitutional isomeric structures*

The case of constitutional isomeric fragments is also worth mentioning. As a typical example in our study, we refer to quinoline derivatives. Quinoline (2OHL) exhibited superior potency and more efficiency than its isomer; isoquinoline (2OHK) in binding to BACE-1 active site. Our results confirmed that structural isomers may have a determinant effect on both binding energies and efficiencies. In this particular case, both of the isomers show similar enhancement patterns in their potency and efficiency profiles. For further information, some constitutional isomers and their normalized docking-based energies and efficiencies are depicted in Table 3. 

*Side chains in designing potent BACE-1 inhibitors*

For further evaluation of the side chain effect on ligand potency, we focused on identical isophthalamide simplified structures derived from different BACE-1 inhibitors (31-33). Experimental BACE-1 inhibitory activities for a number of crystallographic ligands and binding characteristics of their docked fragments are summarized in Table 4. 

**Table 4:** Experimental IC_50_s for crystallographic BACE-1 inhibitors and structure-based binding indices in related fragments

**PDB code **	**IC**_50_ **s of ** **BACE1 inhibitors ** **(nM) **	**Docked fragments **		
		**BE **	**LE **	**BEI **
1W51	500	-10.55	0.377	20.67
2P83	11	-10.96	0.391	21.47
3L58	15-80	-11.84	0.377	23.20
Priority order	2P83>3L58>1W51	3L58>2P83>1W51	2P83>1W51≈3L58	3L58>2P83>1W51

**Figure 8:**
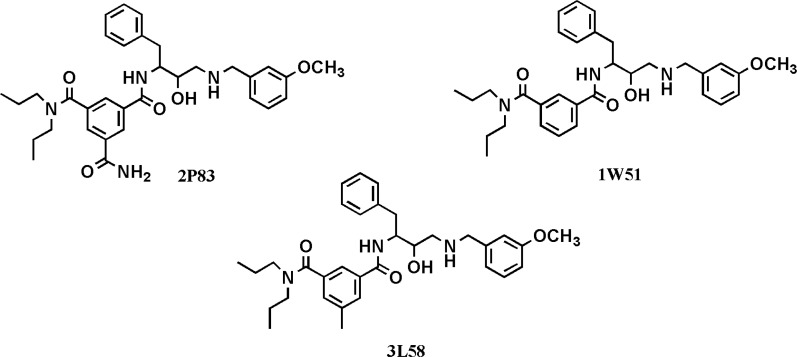
Chemical structures of isophthalamide-based BACE-1 inhibitors with their relevant PDB codes used for the assessment of side chain effects

Experimental data showed that the priority order was not retained in docked fragments due to the absence of side chains. Studied fragments possessed 4 heteroatoms in their structures (2 nitrogen atoms and 2 oxygen atoms) and this may also to some extent explain the dissimilar observed priority orders for LE and BEI values. BE and BEI indices are in good agreement with each other. Based on the results, the well-orientated 3L58 derived fragment is possibly an efficient starting point to develop BACE- 1 inhibitors. Different biological activities of cognate structures could be attributed to the absence or presence of side chains in the *meta *position of isophthalamide ring (Figure 8). It should be added that all the evaluated structures possessed the same stereochemistry. 

These typical cases may be beneficial in quantification studies of side chain effects on binding potency of biologically active molecules. 

## Conclusion

Docking simulation was applied in comparative study of BACE-1 inhibitors. We used a simple shape-based dissection method to analyze compounds obtained from PDB source. This classification route was followed by a fragment-based docking strategy to rank the structural features of BACE-1 inhibitors. The merit of such structure-based efficiency indices for various biological targets has been well revealed in other studies. Our case study confirmed that larger BACE-1 inhibitors may require special attention in their design toward efficiency indices. According to this, considering molecular weights rather than number of heavy atoms would produce less biased results. This technique leads to more realistic view at the individual atom participations in binding to the receptor. Comparative studies revealed that *N*-(4-(benzylamino)-3-hydroxy-1-phenylbutan- 2-yl) benzamide could be an appropriate fragment for further bioactive molecular modifications. Other efficient fragments were arylpiperazine, imidazolidinone, pyrimidinone and benzoimidazole. Our study confirmed that the evaluation of the ligand-receptor interactions on the basis of ligand efficiency indices (binding energy per atom and pK_i _per MW) rather than free energy of binding (ΔG) could be a helpful strategy in recognizing potential candidates for further bioactive molecular developments.
